# Adapted motivational interviewing to improve the uptake of treatment for glaucoma in Nigeria: study protocol for a randomized controlled trial

**DOI:** 10.1186/1745-6215-15-149

**Published:** 2014-04-29

**Authors:** Mohammed M Abdull, Clare Gilbert, Jim McCambridge, Jennifer Evans

**Affiliations:** 1 Ophthalmology Department, Abubakar Tafawa Balewa University Teaching Hospital, Hospital Road, PMB 0117 Bauchi, Bauchi State, Nigeria; 2 Department of Clinical Research, London School of Hygiene and Tropical Medicine, Keppel Street, London WC1E 7HT, UK; 3 Department of Social and Environmental Health research, London School of Hygiene and Tropical Medicine, Keppel Street, London WC1E7HT, UK

**Keywords:** Glaucoma, Motivational interviewing, Africa, Blindness, Treatment adherence, Randomized clinical trial

## Abstract

**Background:**

Glaucoma is a chronic eye disease associated with irreversible visual loss. In Africa, glaucoma patients often present late, with very advanced disease. One-off procedures, such as laser or surgery, are recommended in Africa because of lack of or poor adherence to medical treatment. However, acceptance of surgery is usually extremely low. To prevent blindness, adherence to treatment needs to improve, using acceptable, replicable and cost-effective interventions. After reviewing the literature and interviewing patients in Bauchi (Nigeria) motivational interviewing (MI) was selected as the intervention for this trial, with adaptation for glaucoma (MIG). MI is designed to strengthen personal motivation for, and commitment to a specific goal by eliciting and exploring a person’s reasons for change within an atmosphere of acceptance and compassion. The aim of this study is to assess whether MIG increases the uptake of laser or surgery amongst glaucoma patients where this is the recommended treatment. The hypothesis is that MIG increases the uptake of treatment. This will be the first trial of MI in Africa.

**Methods:**

This is a hospital based, single centre, randomized controlled trial of MIG plus an information sheet on glaucoma and its treatment (the latter being “standard care”) compared with standard care alone for glaucoma patients where the treatment recommended is surgery or laser.

Those eligible for the trial are adults aged 17 years and above who live within 200 km of Bauchi with advanced glaucoma where the examining ophthalmologist recommends surgery or laser. After obtaining written informed consent, participants will be randomly allocated to MIG plus standard care, or standard care alone. Motivational interviewing will be delivered in Hausa or English by one of two MIG trained personnel. One hundred and fifty participants will be recruited to each arm. The primary outcome is the proportion of participants undergoing laser or surgery within two months of the date given to re attend for the procedure. MIG quality will be assessed using the validated MI treatment integrity scale.

**Discussion:**

Motivational interviewing may be an important tool to increase the acceptance of treatment for glaucoma. The approach is potentially scalable and may be useful for other chronic conditions in Africa.

**Trial registration:**

ISRCTN79330571 (Controlled-Trials.com).

## Background

Glaucoma, a chronic eye disease of unknown cause, is responsible for irreversible blindness in roughly 8.4 million people worldwide [1]. In Nigeria, the prevalence of blindness in those aged ≥40 years is 4.2% with 16.3% being due to glaucoma [2]. Glaucoma causes painless, progressive loss of the peripheral field of vision leading to total, irreversible blindness [3]. Primary open angle glaucoma (POAG), which is the most common type in Africa [4], is asymptomatic in the early stages [5]. In Africa, patients with glaucoma present very late, usually with a very advanced stage of the disease [6-11]. In an earlier unpublished study (Abdull MM, Stage at presentation of primary open angle glaucoma in Northern Nigeria) in the same clinic in Bauchi, 75% of the eyes of new glaucoma patients were already blind.

The aim of treatment in glaucoma is to lower the intraocular pressure (IOP), which slows or halts progression [12]. Treatments are daily eye drops, surgery or laser. Surgery and laser are one-off procedures, and laser can be repeated. Both have acceptably high rates of success. Eye drops (used daily for life) are not recommended in Africa except for educated people who live near eye units, as adherence in other groups is very low. Surgery is the recommended treatment for glaucoma in Africa [13-16]. However, acceptance of surgery can also be extremely poor. In the earlier unpublished study carried out two years before the trial was planned, fewer than 5% of people offered surgery (trabeculectomy) returned for the procedure. Laser treatment was not available at that time. To prevent glaucoma blindness it is therefore necessary to improve acceptance and adherence to treatment using approaches that are acceptable, replicable and cost-effective in the African setting.

### Motivational interviewing

A review of the literature on approaches to improve adherence to treatment of any kind, and findings from qualitative research in Bauchi were used to modify a form of counseling, called motivational interviewing (MI) [17]. Motivational interviewing is designed to strengthen personal motivation for and commitment to a specific goal by eliciting and exploring the person’s own reasons for change within an atmosphere of acceptance and compassion. It has shown promise in psychiatry, substance abuse, and healthy life style changes and is being increasingly used in other fields of medical and health care [18-20]. It is an approach that can be taught at most levels [21,22], although it is complex to learn and training workshops alone are insufficient. We have named this modified form of MI as MI modified for glaucoma (MIG) for the purpose of this study.

A Cochrane review of interventions for improving adherence to eye drops for glaucoma did not find evidence to support any particular method [23]. None of the studies included in the review were undertaken in Africa and there is no review of acceptance of surgery or laser treatment. Other Cochrane reviews show the effectiveness of MI for other conditions [24,25].

To our knowledge this will be the first trial of this nature to be undertaken in Africa. Similar studies in the United Kingdom and United States have only assessed adherence to medical treatment [26,27].

There is a need for a strategy that is both feasible and effective in increasing awareness about the disease and its management, and the benefits of treatment, to improve acceptance. MIG is a relatively inexpensive technology and local people can be trained to deliver it in the local language. The pilot study described below demonstrated that MIG is acceptable to patients who are not literate and who have no or little knowledge of glaucoma. We acknowledge that there are difficulties in learning and applying this complex approach in the resource-constrained situation in Nigeria, where exposure to counselling of any type is unusual, and where a high proportion of the population have not received any formal education. Bearing this in mind, the two interviewers who will deliver the intervention come from the same community as study participants and are familiar with local social constructs, customs, beliefs and communication patterns, and are also bilingual in English and the predominant local language, Hausa.

The quality of counselling is important in all studies of this type. The Working Alliance Inventory questionnaire (WAI) [28] has been developed to assess the perceptions of both the counsellor and the participant. In this trial, WAI questionnaires will be completed immediately after the MIG session and analyzed as in other studies [29,30]. Recorded interviews will also be assessed for fidelity by independent experts, using the motivational interviewing treatment integrity (MITI) scale, a validated tool used for the fidelity testing of MI [31].

### Pilot study October 2012 to March 2103

The purpose of the pilot study was to assess the acceptability and quality of MIG in adults with advanced glaucoma, to refine the study protocol and finalize data forms, to provide data for the sample size calculation, to refine recruitment and randomization processes and to estimate recruitment rates. The primary outcome was acceptance of laser or surgery on the date given, which was around one month after the date of diagnosis, recruitment and randomization.

### Findings of the pilot study

All those eligible to be included in the pilot study agreed to take part, approximately 20 eligible patients were recruited each month, and the method of randomization using random numbers in sealed envelopes gave equal numbers allocated to MIG or no MIG. All who were offered a MIG interview accepted, and interviews took 20 to 30 minutes per session. None of those offered a second session returned and some participants preferred to have a relative or companion with them. None found the process distressing and reviewing the transcripts showed the quality of the MIG sessions to be satisfactory, but with room for improvement. Data on the primary outcome were available on 45 participants (Table 1). Nine of the nineteen (47%) participants who had undergone MIG underwent treatment compared with nine of the twenty six participants (35%) who had not undergone MIG. MIG therefore increased treatment rates by 12%. Overall acceptance was 40%, and 75% of these participants underwent laser treatment over surgery. No participants attended for surgery after the date given.

**Table 1:** Pilot study

		**Had surgery**			
		**Yes**	**No**		
**MIG**	Yes	9	10	19	47.4%
	No	9	17	26	34.6%
		18	27	45	

MIG, motivational interviewing modified for glaucoma.

We concluded that MIG is acceptable and the time interval for the primary outcome can be reduced from acceptance within four months of the date given for surgery or laser treatment, to acceptance within two months, as all those accepting treatment did so on the date given and none returned at a later date. Laser treatment is deemed to be more acceptable than surgery.

## Methods/Design

The primary hypothesis is that MI, locally adapted for glaucoma and its treatment (MIG), increases the uptake of treatment amongst individuals with advanced glaucoma in Bauchi State, Nigeria.

A randomized controlled trial with 1:1 allocation to intervention or no intervention will be used to test this hypothesis.

### Study setting

This is a single centre trial, taking place at Abubakar Tafawa Balewa University Teaching Hospital (ABUTH), Bauchi, Bauchi State, Nigeria. Dr M Abdull is the senior ophthalmologist. The eye department is new and has recently been re-equipped and additional staff appointed.

### Participant eligibility

#### Inclusion criteria

Inclusion criteria for the trial are as follows: must be aged 17 years or above, have a confirmed diagnosis of POAG, have surgery or laser agreed to be the best option for further treatment, are able to understand Hausa or English, and must live within 200 km of the clinic.

#### Exclusion criteria

Exclusion criteria for the study are as follows: patient does not consent, there are other ocular morbidities, any diagnosed systemic diseases that contraindicate surgery or laser, communication problems (such as profound deafness), previous eye surgery (except cataract surgery), or have been referred to Abubakar Tafawa Balewa University Teaching Hospital (ATBUTH) specifically for glaucoma surgery.

### Identification of potential participants and recruitment

Potential participants will be those with POAG where the examining ophthalmologist recommends surgical treatment (trabeculectomy with or without anti-scarring agents) or laser (diode laser trans-scleral cycloablation treatment of the ciliary body which is being increasingly used for advanced glaucoma) [32,33]. Patients with POAG will be identified by screening everyone aged 17 years and above who attends the outpatient department, regardless of their presenting complaint(s) (Additional file 1). Standard clinical procedures will be used to detect people who the ophthalmic nurse or optometrists suspect as having glaucoma, who will then be examined by an ophthalmologist to confirm the diagnosis. Criteria for referral to the ophthalmologist are one or more of the following:

A cup disc ratio of 0.7 or more in one or both eyes. Optic discs will be assessed in all patients by direct ophthalmoscopy through undilated pupils in a dark room. If the disc is not visible, the Van Herrick’s test will be carried out to exclude narrow angles. The pupils will be dilated and optic discs examined at the slit lamp using a +60D lens; Cup disc ratio difference of 0.2 or more between the two eyes. Examination as before. The cup disc ratio of the two eyes will be compared to detect differences of 0.2 or more; Positive family history of glaucoma regardless of the eye findings. In the Rotterdam study there was a 9.2 relative risk for individuals with a family history of glaucoma [34]. In the Tasmania study the odds ratio of having a positive family history of POAG was 4.1 [35]. A person has a 20% risk if a parent has the disease, increasing to 50% for a sibling; IOP greater than 26 mmHg in the absence of a view of the discs, even after dilated examination, measured by Goldman application tonometry using standard techniques; Relative apparent pupillary defect (RAPD) assessed in a darkened room using the swinging flash light test [36]; and high myopia or history of distance spectacle use because of the association with glaucoma [37].

Everyone suspected of having glaucoma will be referred to the ophthalmologist (usually Dr Abdull, but other ophthalmologists will also be trained to detect those who are eligible) for a detailed routine ophthalmic examination. The ophthalmologist will confirm the diagnosis of POAG and determine the treatment of choice based the clinical findings (severity) and socio-economic factors likely to influence adherence to medical treatment (such as education and distance from the hospital). Long-term eye drops will be recommended for those who live near the hospital, are educated and can afford topical medication. These individuals will not be eligible for recruitment. Surgery or laser treatment will be recommended for those with an advanced stage of the disease where this offers the best hope for preventing blindness. These individuals will be potential participants. The ophthalmologist will then explain the disease, the treatment options, and the purpose of treatment, as is standard of care. Participants will choose whether to have laser treatment or surgery. The ophthalmologist will then prescribe eye drops and explain how they are to be used whilst waiting for surgery or laser treatment. Everyone offered surgery or laser treatment will be given a date within one month to re-attend for the procedure. Their name and hospital registration number will be written in the surgical register. They will then be escorted to the project manager for recruitment. Participants wishing to change their date of treatment will be offered a new date, and the surgical register, data record form and spreadsheet will be updated.

The eligibility of each potential participant will be checked by the project manager. All those eligible will be recruited after obtaining written informed consent. If the patient does not consent, reasons will be sought and recorded. After obtaining consent a unique identifier number will be issued. All those recruited will be given a red ID card which contains their name, hospital number and study ID. They will be required to present this card at the registration desk at every visit.

### Randomization

The randomization list was generated in Excel, using the *rand between* function by Jennifer Evans, away from the project site. Block randomization with a variable block size was used to ensure that the groups will be balanced over time, as the uptake of laser treatment or surgery may fluctuate over time.

The option of MIG or no MIG was printed on headed paper which was signed and stamped. Each was placed in sequentially-numbered opaque envelopes according to the randomization schedule. Each envelope was sealed and stamped. The same was done for randomization to interviewer A or B. These procedures were undertaken in London by persons not involved in the trial.

For each participant, the interviewer in Bauchi will take the next envelope in the sequence and open it to see whether the patient is allocated to MIG or not. They will write the sequence number on the participant’s form and their unique ID number on the outside of the envelope. After returning the letter to the envelope, the envelope will be kept in a sealed, locked container. The same process will be followed for allocation to interviewer A or B.

All participants, whether randomized to MIG or not, will be given an educational graphic leaflet called ‘Silent Thief’ (Additional file 2). All participants will, therefore, receive some additional information about glaucoma and its management.

### MIG Intervention

**Figure 1:**
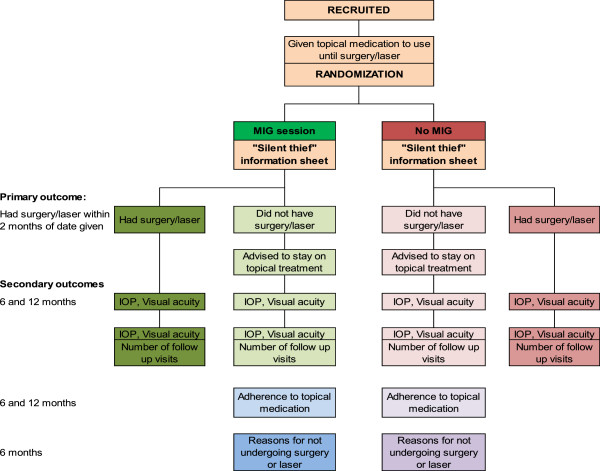
**Randomization Flow chart.** IOP, intraocular pressure; MIG, motivational interviewing modified for glaucoma.

Two trained personnel will deliver MIG sessions (Figure 1).The interview will be conducted in a quiet room within the clinic, in the participant’s preferred language (Hausa or English). Interviewers and participants will be asked to switch off their mobile phones. The participant may be accompanied by someone of their choice.

The interviewers will introduce themselves and explain that the session will be confidential. The interviewer will then try to engage the participant by presenting an agenda for discussion, giving the participant the option to choose where to start. The topics covered will be: awareness of glaucoma and the consequences of no treatment; acceptance of treatment options, especially surgery; adherence to topical medication when prescribed; and need for follow up to monitor the pressure inside the eye.

Open-ended questions will be used, with active listening and reflections. The interviewer will seek to understand the participant’s perspective in an empathic fashion. Participant autonomy will be honored as information on glaucoma and its treatment will only be given if requested, or after gaining permission if the participant seems ready for this. The interviewer will listen for change talk, such as participants showing desire for surgery with terms like ‘I wish to’; or an ability to undergo surgery, with ‘I can’; or a reason for change, such as ‘I need vision to save my job’; or commitment, with terms like ‘I intend to’; or even a statement showing that the participant is already taking steps to come for surgery, ‘I have discussed the need for financial support with my family’. The interviewer responds appropriately with reflections, affirmations, and requests for further elaboration or further evocation. At the end of the session, a summary of the interview will be presented to the participant by the interviewer.

The MIG sessions will seek to follow the spirit of MI, and will be recorded for later fidelity testing. Each interview session will last 30 to 60 minutes. All interview records will be kept in a locked cabinet in the office. After the interview, the WAI Short Questionnaire will be administered to establish if there was rapport and to assess the interviewer and patient’s thoughts about the interview.

Under some circumstances a second MIG session might be required if the participant could not stay for the session on the day of recruitment, if the interview was not completed as the participant had to leave, or if there were frequent interruptions from the companion or others. If, in the opinion of the interviewer, the session could not be conducted as planned, the reasons will be recorded and the participant offered a second session.

### Assessing the quality of the MIG intervention

This will be undertaken in three ways. Firstly, the WAI Short Questionnaire, which is completed by the interviewer and participant immediately after the session, will be used to investigate the relationship between the perceived quality of the interview and trial outcomes (Additional files 3 and 4). Participants who cannot see well, or who cannot read the questionnaire themselves will have the questions read out to them by the other interviewer (to reduce bias). Secondly, a random sample of interviews in English, and translated interview transcripts in Hausa will be sent for independent fidelity testing using the MITI scale, a validated tool used for fidelity testing of MI. Thirdly, taped interviews in Hausa and English will be listened to by Dr Abdull and the interviewers on a regular basis throughout the trial as part of supervision sessions, to discuss how well the sessions are going, to identify issues which arise and learning needs, and how interviewing could be improved.

### Outcomes

#### Primary outcome

The primary outcome will be the proportion of participants undergoing laser treatment or surgery within two months of the date given to attend the hospital for the procedure (Table 2. Participant timeline).

**Table 2:** Participants time line

**Participant timeline:**				
	**T0**	**T1**	**T2**	**T3**
		**Date for surgery/laser + 2 months**	**T0 + 6 months**	**T0 + 12 months**
Diagnosis of glaucoma	X			
Standard explanation of disease/treatment	X			
Prescribed eye drops	X			
Recruitment	X			
Randomization	X			
MIG session	Yes/No			
**Primary outcome assessment:**				
Attended for surgery/laser		Yes/No		
**Secondary outcome assessment:**				
Visual acuity and IOP			X	X
**Other measures:**				
Adherence to topical medication			X	X
Reasons for not undergoing procedure			X	

#### Secondary outcomes

At 6 and 12 months after randomization the following will be assessed and recorded: mean IOP (can be measured by Tonopen in their home, if needed); proportion with loss of three of more lines of visual acuity, or loss of form vision (cannot read any letters on the chart) measured using a LogMar chart. This can be measured in their home, if needed; and mean number of follow-up visits to monitor IOP and other clinical parameters.

### Other data

At 6 months from the date given for laser treatment or surgery, a subset of participants in each arm of the trial who do not undergo laser treatment or surgery will be contacted and asked to return to the clinic. Those who attend will be re-offered treatment; those who do not attend will be visited in their homes. The following data will be collected from all: scores using the Morisky questionnaire to assess adherence to topical medication, and reasons why participants did not undergo surgery or laser in both arms of the trial.

### Sample size calculation

The sample size is based on the primary outcome and calculated using the SAMPSI command in Stata 12.1 statistical software (StataCorp Texas, USA). Based on the pilot study, we anticipate that 35% of participants in the standard care group will undergo surgery or laser within two months of the date given for the procedure. A sample size of 150 in each arm will be required to detect an acceptance rate of 52.5% (50% relative and 17.5% absolute increase in acceptance) in people in the intervention arm (power 0.8, alpha 0.05). This is based on a calculation of 137 in each arm rounded up to 150 in each arm to allow for loss to follow-up. We believe this absolute increase of 17.5% (corresponding to a relative increase of 50% assuming a 35% acceptance in the control group) would be an important effect to detect. We have also calculated the power of a study of this size to detect differences between the treatment groups with respect to the secondary outcomes. IOP at entry to the study will average approximately 35 mmHg (SD +/- 14 mmHg) (values resulting from the pilot study). Our outcome is based on the final IOP at 12 months. This is likely to be in the order of 25 mmHg in the standard care group. The study will have good power (0.84) to detect differences of 5mmHG or more between the two groups at one year.

**Figure 2:**
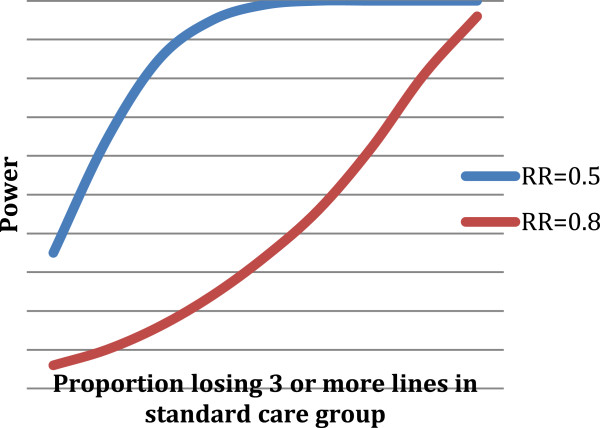
**Proportion of participants losing three or more lines of visual acuity in the standard care group.** RR, Relative risk.

We have less information on the probability of losing three or more lines of visual acuity over the year. Figure 2 shows the proportion of participants losing three or more lines of visual acuity in the standard care group, with power curves for risk ratios of 0.5 and 0.8. It is more likely that we will be able to detect risk ratios in the order of 0.5. The power of the study to detect significant differences using a relative risk of 0.5 is shown in Table 3.

**Table 3:** Power of the study to detect significant differences using a relative risk of 0.5

**% in standard care group losing 3 or more lines of visual acuity over 12 months**	**Power to detect RR of 0.5**
10%	0.35
20%	0.58
30%	0.81
40%	0.94
50%	0.99
60%	1
70%	1
80%	1
90%	1

RR, Relative risk.

### Blinding

Only participants allocated to MIG and the interviewers will know who has been allocated to MIG. The project manager and study ophthalmologist, who will be responsible for obtaining and checking data on the primary outcome, will be blinded to the intervention.

### Ascertainment of outcomes

#### Primary outcome

All data on socioeconomic variables, clinical findings and the date given for surgery will be entered into a customized database on the day of recruitment. Software will be used to generate a spreadsheet which will be constantly updated. The spreadsheet will have the patient’s unique ID, name, sex, hospital number, detailed address, contact phone number, the date given for surgery (or the revised date, if relevant), the date the patient came for surgery and the ‘tracing date’ (date two months after the date given for surgery). The spreadsheet will not contain the randomization sequence number to maintain blinding. The software will be programmed to indicate the date two months after the date booked for laser treatment or surgery (or the revised date). This is the ‘tracing date’ when participants will be traced (see below). Every surgery day the spreadsheet will be reviewed to identify participants who should attend for surgery or laser treatment that day. The surgical register will be checked to see who did and who did not attend on the appointed operation day. The data recording form will be completed. The data will be entered into the database so that the spreadsheet can be updated.

#### Secondary outcomes

These outcomes will be assessed at 6 and 12 months after randomization. Participants may also attend at other time points for clinical reasons. At the 6 month (+/- one month) and 12 month visits (+/- one month), a full clinical examination will be performed on those who return. Data on IOP and visual acuity will be recorded. Participants who do not attend the 6 and/or 12 month visits will be traced, initially by telephone, requesting them to visit. If they still do not attend the clinic, they will be visited in their home, where their visual acuity will be measured using a LogMAR chart, and IOPs measured using a Tonopen.

### Other measures

These will be assessed at 6 and 12 months after randomization. Adherence to topical medication in those not undergoing surgery or laser treatment will be assessed using the Morisky score [14] (Additional file 5). This can be administered over the phone, if necessary. Prior to assessing reasons for not undergoing surgery or laser treatment, the following will be assessed after taking verbal consent: a) whether they did in fact attend for surgery at ABUTH, but slipped through the net; b) they underwent surgery or laser treatment in another hospital, or were given more eye drops elsewhere; and c) they have not had any treatment for their glaucoma apart from the eye drops given at the time of recruitment.

Those who have not had any further treatment will be interviewed to find out why they did not attend. This can be administered over the phone. A subset of those not attending will be selected randomly, and visited in their homes for in-depth interviews where they will be asked why they did not return to the hospital. Responses will be probed to better understand the barriers that led to non-acceptance of laser or surgery. See Additional file 6 for the interview guide and probe questions.

### Data collection

The following data will be collected using pre-tested data forms (Additional file 5).

### Before and after recruitment at T0

Data will be collected for name, age, gender, clinical findings and date given to attend for surgery or laser treatment. For those who refuse, reasons for not agreeing to participate will be sought. For those who agree, contact details will be recorded: detailed address; telephone numbers of individual/family/neighbor; sociodemographic data and randomization sequence number. All data will be entered into a customized, password protected database on the day of recruitment. The form will be kept in a lockable cupboard only accessible to the project manager in an office dedicated to the trial, which is locked when not in use.

For those allocated to MIG the interview will be conducted at T0. If a second MI session is indicated, the reason for this will be recorded. After every interview, WAI questionnaires will be completed independently by the participant and the interviewer. These forms will be kept in a lockable cupboard in a separate office dedicated to the MIG interviews. This office will be locked at all times when not in use for MIG sessions. The project manager and ophthalmologist will not have access to these forms at any time. Data from these forms will be entered into a separate password protected database.

### At time of ascertainment of primary outcome T1

Information on whether the participant underwent laser or surgery, which procedure was performed, and the date of the procedure will be recorded.

### At time of ascertainment of secondary outcomes T2 and T3

The date attended and the IOP and visual acuity in the study eye will be recorded. The Morisky Adherence questionnaire will be administered. At T2 only, the reasons for not undergoing surgery or laser will be sought using closed-ended questions. Consent will be obtained to record all in-depth interviews. (See Additional file 6 for interview guide for in-depth interviews.)

### Data management

#### Data entry

Databases have been created in Epidata and Access, with range and consistency checks. Data will be double entered by the project manager, who has been trained in data entry. Data will be entered as soon as possible after recruitment, so that the ‘surgery date’ and ‘tracing date’ outputs can be generated (as above). Random checks of the quality of data entry will be undertaken by Dr Abdull on a regular basis. Frequency distributions will be explored and data of outliers checked for accuracy.

### Data analyses

The randomization code will only be broken once analysis of the primary outcome is completed. We will prepare a flow chart describing participant flow through the trial. This diagram will provide data on the following: the number of eligible people approached to take part in the trial, the number of people who agreed to take part, reasons for non-participation, the number of people randomly allocated to MIG and no MIG, the number of people who received the intervention as randomized, the number of people with data on the primary outcome by the intervention group, and the number of people followed up at 6 and 12 months by the intervention group.

We will compare people who agreed to take part in the trial with people who did not agree to take part in terms of age, sex, education and stage of glaucoma to assess the generalizability of the findings. All participants will be analyzed in the group to which they were randomized (by intention to treat).

We will compare the characteristics of the people in the two intervention groups with respect to age, sex, education, distance from hospital and stage of glaucoma at presentation to assess the balance between intervention groups.

We will describe the quality of the MIG in two ways. Firstly, by the WAI questionnaire scores for participants’ and interviewers’ perceptions of the MIG sessions. We will analyze this as a continuous variable but it is likely that the data will be skewed and we will therefore present medians, ranges and interquartile ranges, by interviewer. We will also compare this to other published data on WAI scores. Secondly, by fidelity testing using the MITI scale, which generates a series of scores that can be categorized as good or poor. We will present results by interviewer as done in published studies using the MITI.

### Primary outcome

Our primary outcome is dichotomous (did or did not attend surgery or laser treatment within two months of their scheduled date). Our effect measure will be the risk ratio, that is, we will calculate the proportion of people with this outcome in the intervention group compared to the standard care group. We will report this with 95% confidence intervals. In a trial of 300 people we anticipate that the groups will be fairly well balanced. However, we will also calculate a risk ratio adjusted for factors that may affect uptake of surgery (stage of glaucoma, age, sex, education, and distance from hospital).

### Secondary outcomes

We will analyze the dichotomous secondary outcomes in the same way. For the continuous secondary outcomes we will calculate the mean difference with 95% confidence intervals, if the data are reasonably normally distributed. Otherwise we will compare the two groups using the median value and assess the role of chance using non-parametric tests.

### Subgroup analysis

We plan two subgroup analyses of the primary outcome. We will calculate the primary outcome risk ratio and do a test for interaction in the following subgroups: Interviewer A versus interviewer B, and ‘Good’ versus ‘Poor’ sessions according to the MITI scale (see above). We will tabulate reasons for not attending for surgery or laser treatment or follow-up by intervention group and the Morisky adherence scores by intervention group.

Consolidated Standard of Reporting Trials (CONSORT) guidelines will be used when reporting results.

### Qualitative data

Interview recordings in Hausa will be transcribed and translated into English, if required. Transcripts will be coded using N vivo (QSR International, Victoria, Australia) and analyzed to identify reasons why individuals did not undergo the treatment recommended and to explore barriers.

### Data monitoring

A Data Management Committee will be established, chaired by an independent clinical trialist. Membership to be confirmed. Stopping rules will not be required, as the intervention is acceptable and is unlikely to cause harm. The pilot study has also demonstrated that MIG is of a modest benefit. Interim analyses will not be undertaken. Meetings will be held regularly throughout the trial (every 6 months) to assess progress and advise if problems arise, and to assist in interpreting the results. Additional meetings will be called if required.

### Ethics and dissemination

Ethical approval has been obtained from the Interventions Ethics Committee of the London School of Hygiene & Tropical Medicine, and from the Institutional Review Board of ATBUTH, Bauchi.

### Protocol amendments

Important protocol amendments will be communicated to the Data Management Committee, the Interventions Ethics Committee of the London School of Hygiene and Tropical Medicine, the trial register, and will be reported in publications and reports.

### Consent

Written informed consent will be obtained by the project manager. The information sheet and consent form will be available in Hausa and English. For those who cannot see well enough to read, or who cannot read, the information sheet will be read out and they will sign or provide a thumb print, which will be witnessed by the project manager. Additional consent will be obtained from those selected for in depth interviews. Specific consent will be obtained to record the interview, and for any anonymous quotes to be used.

### Confidentiality

The names, ID and hospital number of those taking part in the trial will only be known to project staff. Study ID numbers will not be entered into the surgical register. As the surgery date and the date to start tracing individuals for primary outcome data requires names and hospital numbers, these will be need to be entered into the database.

Analysis of all the outcome data will be undertaken after removing all identifiers from the database and any quotes taken from the in depth interviews will use anonymous codes.

### Access to data

The following individuals will have access to trial data: Clare Gilbert, Dr Abdull and Jennifer Evans.

### Post-trial care

Standard clinical care will be provided to all study participants. No adverse events are anticipated.

## Discussion

The trial is designed to assess the effectiveness of MIG in Nigeria in encouraging patients to accept surgery or laser treatment. The MI has been adapted because it is being undertaken in a language other than English and in a different culture where counselling is not the norm; it may be difficult to achieve the proficiency possible in a western audience. In the interview, questions, reflections and providing information are done in strict adherence to the spirit of MI. Interviews are conducted in the local language (Hausa), however participants who understand English may wish to be interviewed in English. At present, the fidelity assessment of motivational interviews using the MITI scale is conducted in English, but after discussion with the assessor it has been agreed that transcribed and translated interviews will also be used for assessment. However, transcripts lead to loss of vital information such as tone of voice, pauses and empathetic sounds. Assessing only interviews conducted in English may lead to bias, as participants who speak English are likely to be better educated and more aware of glaucoma and its treatment.

The initial plan was to offer participants the option to come back for a second MIG session if they so desired, however, in the pilot study no-one returned for a second interview.

During the pilot study the duration of the interviews increased over time as the interviewers gained both experience and confidence. In the main trial participants will be told that the interview will last up to 60 minutes. The longer time will allow participants to ask more questions and so gain greater understanding of the decision they are being asked to make.

The criteria used for identifying glaucoma patients follow a guide by Foster *et al.*[38] and the values for cup: disc ratios and IOP come from the normative dataset of the Nigeria blindness and low vision survey (unpublished data (Kyari F, Normative data for glaucoma in Nigeria. Results from the National Blindness and Low Vision Survey Project)). A limitation of our study is that visual field testing to aid detection of glaucoma is not feasible in a busy eye clinic. The criteria being used may miss some early glaucoma cases, but these individuals would not be eligible for the trial.

The sample size was calculated assuming that 35% of people in the control group will accept surgery. This estimate was obtained from a small pilot study and therefore may be unreliable. If the true acceptance in the control group is 30% then the study will be underpowered to detect a risk ratio of 1.5 (power = 0.69), however it will have good power to detect marginally larger risk ratios of 1.6 or more (corresponding to an absolute increase of 18% of more) (power = 0.84). If the true acceptance in the control group is higher, for example, at 40% then the study will have good power to detect a risk ratio of 1.5 (power = 0.90) (corresponding to an absolute increase of 20% or more). The planned sample size is feasible given the time and resources available for the study and the estimate of acceptance from the pilot study is the best that is available. With the present sample size, subgroup analyses may not be adequately powered to make conclusive inferences.

MI has the potential to be taken to scale in Nigeria and other limited-resource settings, as pre-existing skills in counselling are not required. Indeed, it has been suggested that counselling-naïve individuals are better at MI than those with previous experience, as the main qualities required are empathy, good listening skills and patience. MI also has the potential to be used for other eye conditions where uptake is known to be poor (such as cataract surgery, or lid surgery for trachoma cases) or for other non-ocular conditions.

## Trial status

Recruitment started on 2 September 2013. By the end of January 2014, 70 participants had been recruited, and only one of those eligible has refused.

## Abbreviations

ATBUTH: Abubakar Tafawa Balewa University Teaching Hospital; CONSORT: Consolidated Standard of Reporting Trials; IOP: Intraocular pressure; MI: Motivational interviewing; MIG: Motivational interview for glaucoma; MITI: Motivational interviewing treatment integrity scale; POAG: Primary open angle glaucoma; RAPD: Relative afferent pupillary defect; RR: Relative risk; VCDR: Vertical cup-disc ratio; WAI: Working alliance inventory.

## Competing interests

The authors declare that they have no competing interests.

## Authors’ contributions

MA: Conception and design; data collection; manuscript writing and final approval of the manuscript. CG: Conception and design; revising draft for important intellectual content; final approval of the manuscript. JE: Design; revising draft for important intellectual content; final approval of the manuscript; JMcC: Design; revising draft for important intellectual content final approval of the manuscript; All authors read and approved the final manuscript.

## Supplementary Material

Additional file 1 Randomization flowchart: Flow chart of activities. Click here for file

Additional file 2 Glaucoma the silent thief: Educational material. Click here for file

Additional file 3 Working alliance inventory for interviewer: Questionnaire. Click here for file

Additional file 4 Working alliance inventory for patient: Questionnaire. Click here for file

Additional file 5 Main data record form: Main form for data capture. Click here for file

Additional file 6 Interview guide: Interview guide for those who fail to attend for surgery/laser. Click here for file
