# Supplementary material for: Adapted motivational interviewing to improve the uptake of treatment for glaucoma in Nigeria: study protocol for a randomized controlled trial
Source: Trials. 2014 Apr 29;15:149. doi: 10.1186/1745-6215-15-149 (PMC4021714; doi:10.1186/1745-6215-15-149)
Supplement: Additional file 1 — Randomization flowchart: Flow chart of activities. [file 1745-6215-15-149-S1.pdf]

OPTOMETRIST

Snellen Visual a

Screen for glauc

Compete Screenin

Not a glaucoma suspect

Continue usual care

Not glaucoma

Continue usual care

Not eligible

Continue usual care

Note: If the hospital cancels surgery, and a new date for surgery is given, the **"clock starts again"** and the two month deadline worked out again

Refuse to participate

Complete "reasons why refuse"

Not randomised to MIG
Give "Silent Thief of Sight"
Check that a follow up appoinment has been given for 1 month
Say goodbye

ON 1st POST SURGERY DAY

ON TRACING DATE

FOLLOW UP

NURSE

acuity

coma

ng form

Glaucoma suspected

Take to ophthalmologist

OPHTHALMOLOGIST

Detailed examination for glaucoma

Send for visual fields and disc photos

VISUAL FIELD TESTERS

Visual fields, LogMAR VA disc photos

Print out visual fields. Add date and name

OPHTHALMOLOGIST

Confirm that eligible to be recruited

Select study eye

Give date for surgery. Inform patient

Enter name in surgery book

Briefly explain that the hospital is doing a study

Take to Project Manager with eligibility form

PROJECT MANAGER

Enroll: give/read out information sheet.

Take consent: keep a copy; give participant a copy

Agree to participate

Issue a unique study number

Enter data into Excel spreadsheet

Return participant to ophthalmologist

Complete Section 2 of MAIN Form

Enter surgery date and tracing dates in Excel

Accompany to ophthalmologist

OPHTHALMOLOGIST

Complete Section 1 and Section 3 of MAIN FORM

Accompany to interviewers

INTERVIEWERS

Check name of participant against forms

Check Study no is written on ALL forms & visual fields

Randomization to MIG or no MIG

Take next **brown** envelop: write study no & name on it

Enter sequence number in Section 1 of MAIN Form

Open the envelop. Note if MIG or not

Put envelop in sealed container

Randomization to interviewer A or B

Take next **white** envelop: write study no & name on it

Open the envelop. Note which interviewer

Put envelop in sealed container

Perform MIG: first session

Complete the "working alliance inventory for MI" form for both interviewer and patient

Give "Silent Thief of Sight"

Check date given for surgery: confirm with participant

Check name/date/study no in surgey book

Give date for second session if required

Perform MIG: second session

Complete the "working alliance inventory for MI" form for both interviewer and patient

ASSESSING TRIAL OUTCOME

PROJECT MANAGER / Dr Abdull

Update surgery date and tracing date, if needed

Check whether patient had surgery on surgery date

If yes, complete Section 4 of main form

IF NO - WAIT UNTIL TRACING DATE

On tracing date, call to see if they have had surgery

Complete lower parts of section 1 and 4

If they have not had surgery ask why not

If they have not had surgery and still want it, ask them to return to the hospital for reassesment

Examine patient and fill section 5 for follow up in 1,2,4,6 and 12 months respectively
