# Supplementary material for: Adapted motivational interviewing to improve the uptake of treatment for glaucoma in Nigeria: study protocol for a randomized controlled trial
Source: Trials. 2014 Apr 29;15:149. doi: 10.1186/1745-6215-15-149 (PMC4021714; doi:10.1186/1745-6215-15-149)
Supplement: Additional file 2 — Glaucoma the silent thief: Educational material. [file 1745-6215-15-149-S2.docx]

| **Glaucoma – The Silent Thief of Sight**  Progressive damage (constriction) of your field of vision | | | |
| --- | --- | --- | --- |
| **Normal**  **(full visual field)** | **Moderate visual field loss** | **Severe visual field loss**  **(tunnel vision)** | **Blind** |
| 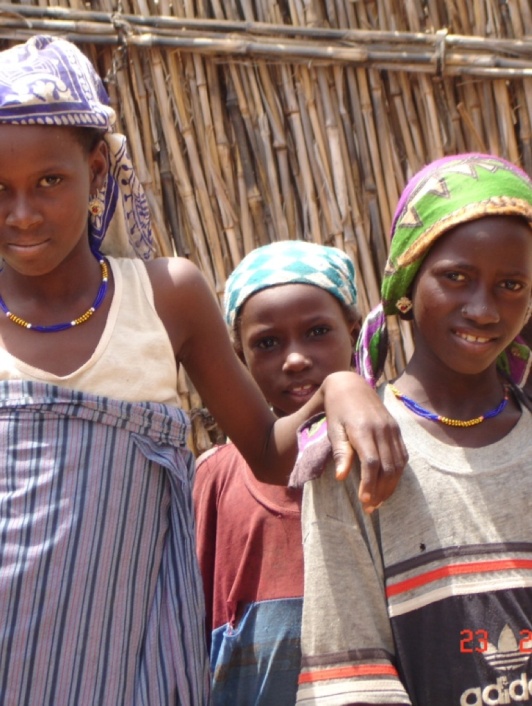 | 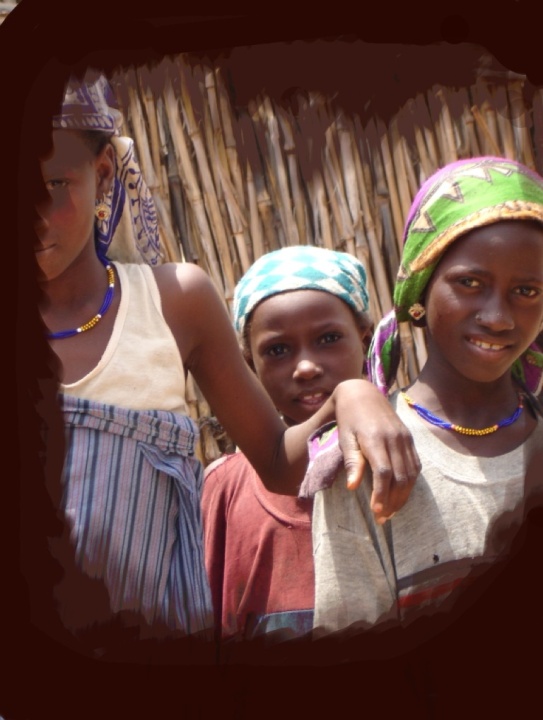 | 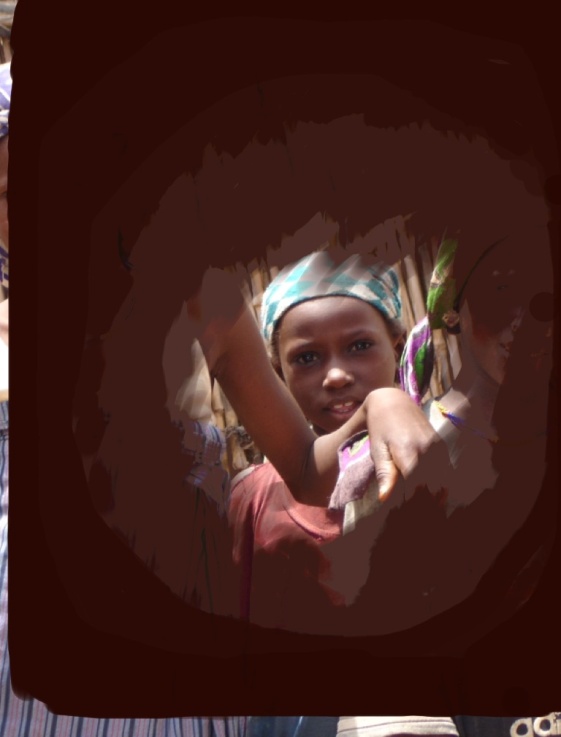 | 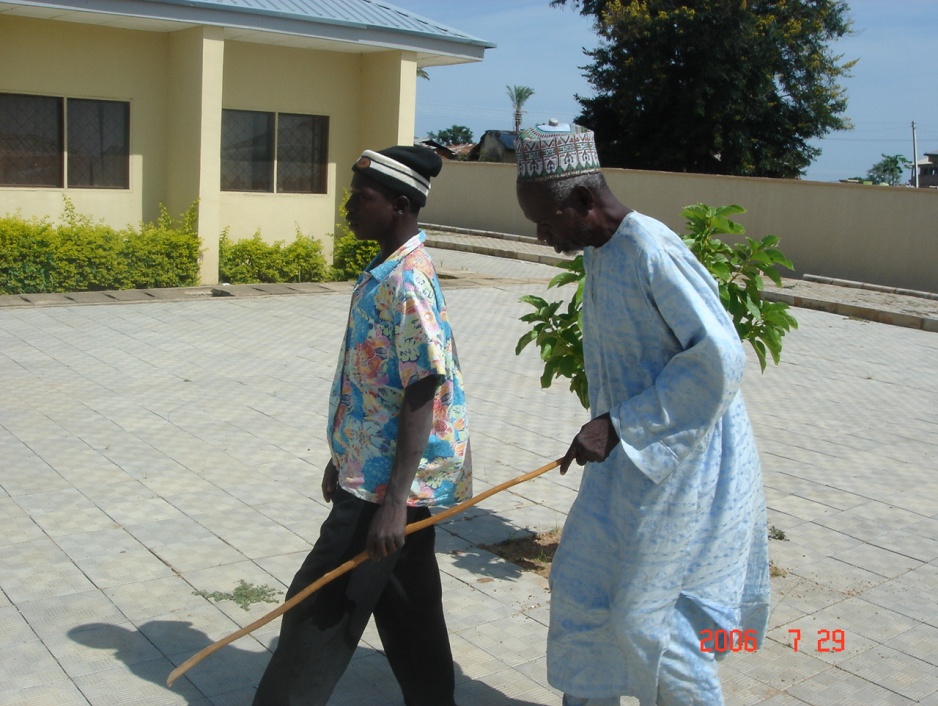 |
| ***Don’t allow this thief to steal your sight:***  ***accept the treatment recommended;***  ***do not delay in accepting treatment;***  ***use your medication as prescribed and***  ***attend for regular follow up*** | | | |
