# Supplementary material for: Adapted motivational interviewing to improve the uptake of treatment for glaucoma in Nigeria: study protocol for a randomized controlled trial
Source: Trials. 2014 Apr 29;15:149. doi: 10.1186/1745-6215-15-149 (PMC4021714; doi:10.1186/1745-6215-15-149)
Supplement: Additional file 3 — Working alliance inventory for interviewer: Questionnaire. [file 1745-6215-15-149-S3.pdf]

Randomised Controlled Trial of Adapted Motivational Interviewing for Acceptance and Adherence to Treatment in Glaucoma Patients in Bauchi

Working Alliance Inventory- Interviewer

**Study number**							
**Hospital registration number**							
**Interviewer ID**							
**Date**							
		Day		Month		Year	
**Measurement Point (circle one):**	1st MIG					2nd MIG	

On the following page there are sentences that describe some of the different ways you might think or feel about your patient.
As you read the sentences mentally insert the name of your patient in place of ________ in the text. Below each statement there is a seven point scale:

1	2	3	4	5	6	7
Never	Rarely	Occasionally	Sometimes	Often	Very Often	Always

If the statement describes the way you *always* feel (or think) circle the number 7; If it never applies to you, circle the number 1. Use the numbers in between to describe the variations between these extremes.
Work quickly, your first impressions are the ones we would like to see.
PLEASE DON'T FORGET TO RESPOND TO EVERY ITEM.
Thank You

1 _______________ and I agree about the steps to be taken to improve his situation.

1	2	3	4	5	6	7
Never	Rarely	Occasionally	Sometimes	Often	Very Often	Always

2 My client and I both feel confident about the usefulness of our current activity in counseling.

1	2	3	4	5	6	7
Never	Rarely	Occasionally	Sometimes	Often	Very Often	Always

3 I believe _______________ likes me.

1	2	3	4	5	6	7
Never	Rarely	Occasionally	Sometimes	Often	Very Often	Always

4 _________________have doubts about what we are trying to accomplish in counseling.

1	2	3	4	5	6	7
Never	Rarely	Occasionally	Sometimes	Often	Very Often	Always

5 I am confident in my ability to help _______________.

1	2	3	4	5	6	7
Never	Rarely	Occasionally	Sometimes	Often	Very Often	Always

6 We are working towards mutually agreed upon goals.

1	2	3	4	5	6	7
Never	Rarely	Occasionally	Sometimes	Often	Very Often	Always

7 I appreciate _______________ as a person.

1	2	3	4	5	6	7
Never	Rarely	Occasionally	Sometimes	Often	Very Often	Always

8 We agree on what is important for _______________ to work on.

1	2	3	4	5	6	7
Never	Rarely	Occasionally	Sometimes	Often	Very Often	Always

9 _______________ and I have built a mutual trust.

1	2	3	4	5	6	7
Never	Rarely	Occasionally	Sometimes	Often	Very Often	Always

10 _______________ and I have different ideas on what his real problems are.

1	2	3	4	5	6	7
Never	Rarely	Occasionally	Sometimes	Often	Very Often	Always

11 We have established a good understanding between us of the kind of changes that would be good for ________________.

1	2	3	4	5	6	7
Never	Rarely	Occasionally	Sometimes	Often	Very Often	Always

12 _______________ believes the way we are working with her problem is correct.

1	2	3	4	5	6	7
Never	Rarely	Occasionally	Sometimes	Often	Very Often	Always
