# Supplementary material for: Adapted motivational interviewing to improve the uptake of treatment for glaucoma in Nigeria: study protocol for a randomized controlled trial
Source: Trials. 2014 Apr 29;15:149. doi: 10.1186/1745-6215-15-149 (PMC4021714; doi:10.1186/1745-6215-15-149)
Supplement: Additional file 4 — Working alliance inventory for patient: Questionnaire. [file 1745-6215-15-149-S4.pdf]

Randomised Controlled Trial of Adapted Motivational Interviewing for Acceptance and Adherence to Treatment in Glaucoma Patients in Bauchi

Working Alliance Inventory- Patient

Name ____

Study number [ ][ ][ ]

Hospital registration number [ ][ ][ ][ ][ ][ ][ ]

Interviewer ID [ ][ ]

Date [ ][ ][ ][ ][ ][ ]
Day Month Year

Measurement Point (circle one): 1st MIG 2nd MIG

Indication for second interview

1	Could not stay for the MIG on day of recruitment
2	Interview not completed as participant had to leave
3	Frequent interruptions from escort
	Other ______________________

Duration of interview [ ][ ] Minutes

Language of interview

1	Hausa
2	English

On the following page there are sentences that describe some of the different ways you might think or feel about your interviewer.
As you read the sentences mentally insert the name of your interviewer in place of ________ in the text. Below each statement there is a seven point scale:

1	2	3	4	5	6	7
Never	Rarely	Occasionally	Sometimes	Often	Very Often	Always

If the statement describes the way you *always* feel (or think) circle the number 7; If it never applies to you, circle the number 1. Use the numbers in between to describe the variations between these extremes.
Work quickly, your first impressions are the ones we would like to see.
PLEASE DON'T FORGET TO RESPOND TO EVERY ITEM.
Thank You

1 ________________ and I agree about the things I will need to do in counseling to help improve my situation

1	2	3	4	5	6	7
Never	Rarely	Occasionally	Sometimes	Often	Very Often	Always

2 What I am doing in counseling gives me new ways of looking at my problem.

1	2	3	4	5	6	7
Never	Rarely	Occasionally	Sometimes	Often	Very Often	Always

3 I believe _______________ likes me.

1	2	3	4	5	6	7
Never	Rarely	Occasionally	Sometimes	Often	Very Often	Always

4 _______________ does not understand what I am trying to accomplish in counseling.

1	2	3	4	5	6	7
Never	Rarely	Occasionally	Sometimes	Often	Very Often	Always

5 I am confident in _______________ 's ability to help me.

1	2	3	4	5	6	7
Never	Rarely	Occasionally	Sometimes	Often	Very Often	Always

6 _______________ and I are working towards mutually agreed upon goals.

1	2	3	4	5	6	7
Never	Rarely	Occasionally	Sometimes	Often	Very Often	Always

7 I feel that _______________ appreciates me.

1	2	3	4	5	6	7
Never	Rarely	Occasionally	Sometimes	Often	Very Often	Always

8 We agree on what is important for me to work on.

1	2	3	4	5	6	7
Never	Rarely	Occasionally	Sometimes	Often	Very Often	Always

9 _______________ and I trust one another.

1	2	3	4	5	6	7
Never	Rarely	Occasionally	Sometimes	Often	Very Often	Always

10 _______________ and I have different ideas on what my problems are.

1	2	3	4	5	6	7
Never	Rarely	Occasionally	Sometimes	Often	Very Often	Always

11 We have established a good understanding of the kind of changes that would be good for me. _______________.

1	2	3	4	5	6	7
Never	Rarely	Occasionally	Sometimes	Often	Very Often	Always

12 I believe the way we are working with my problem is correct.

1	2	3	4	5	6	7
Never	Rarely	Occasionally	Sometimes	Often	Very Often	Always
