# Supplementary material for: Adapted motivational interviewing to improve the uptake of treatment for glaucoma in Nigeria: study protocol for a randomized controlled trial
Source: Trials. 2014 Apr 29;15:149. doi: 10.1186/1745-6215-15-149 (PMC4021714; doi:10.1186/1745-6215-15-149)
Supplement: Additional file 6 — Interview guide: Interview guide for those who fail to attend for surgery/laser. [file 1745-6215-15-149-S6.docx]

**APPENDIX G. Interview guide for those failing to attend for surgery/laser**

Interviewed alone or with family member / neighbour (indicate as necessary)

Greeting and introduction.

I expect you can remember that a few months ago you visited ATBUTH Eye Department where you were found to have the eye condition called glaucoma. At the time you were offered a date to come back for treatment, but until now you have not attended the hospital.

I would be grateful if you could let me know what the reasons were for this

**Possible probe questions:**

| Could not afford the treatment | - Could you afford the travel costs? - How much could you afford to pay? - How might the money be found for you to have the eye treatment recommended? - Who in the family makes the financial decisions for medical or hospital treatment? - What health conditions in the family take priority if money is limited? - If the treatment were subsidised or free, would you accept surgery? |
| --- | --- |
| Fear of the procedure | - Can you tell me what it is you fear the most about the treatment for your eyes? - Do you know of anyone else who has had hospital treatment for an eye condition? If so, has that influenced your decision? |
| No-one to accompany them to hospital | - Who usually accompanies members of your family to seek help when they become ill? - Were you given any reasons why they could not accompany you to have treatment for your eye complaint? |
| Not considered a priority | - Do you have other health problems yourself which are more important than your eye condition? If so, what are they? In what way are they more important - Do other family members have health problems which are more important than your eye condition? If so, what are they? |
| Prefers other forms of treatment e.g. spiritual / herbal | - Have you sought an opinion from anyone else in the meantime? - If so, who have you consulted? - What did they recommend? - Have you accepted their advice or treatment? - Are you satisfied with the advice or treatment you have received? |

I would now like to ask you what you think is the cause of your glaucoma?

Is there anything else you would like to tell me about your eye condition?

If you would still like to have treatment for your glaucoma this can easily be arranged. I can arrange a date for you and let you or a family member know which date it is by text, if this is convenient.

Thank you for your time
